# Increased intra-myometrial vascularity adds diagnostic value to MRI for high-risk placenta accreta spectrum

**DOI:** 10.1038/s41598-025-33179-0

**Published:** 2025-12-22

**Authors:** Hainan Ren, Weichun Liu, Li Zhu, Yang Song, Lijun Qian, Jianrong Xu, Yan Zhou

**Affiliations:** 1 Department of Diagnostic Radiology, Shanghai Jiao Tong University School of Medicine Affiliated Renji Hospital, Shanghai, China; 2 Department of Obstetrics and Gynecology, Shanghai Jiao Tong University School of Medicine Affiliated Renji Hospital, Shanghai, China; 3 MR Scientific Marketing, Siemens Healthineers Co Ltd, Shanghai, China; 4 Radiology department, Renji Hospital, No. 160 Pujian Road, Pudong, Shanghai, 200127 China

**Keywords:** High-risk PAS, MRI, Increased intra-myometrial vascularity, Abnormal vascularization, Bleeding, Anatomy, Diseases, Health care, Medical research, Risk factors

## Abstract

Accurate prenatal diagnosis of high-risk placenta accreta spectrum (PAS) is vital for maternal safety and can mitigate severe complications. We aimed to investigate the diagnostic value of a novel MRI sign, “increased intra-myometrial vascularity (IIMV),” and to evaluate its contribution to an optimal MRI sign combination for differentiating high-risk placenta accreta spectrum (PAS), specifically placenta increta (PI) and percreta (PP), from placenta accreta (PA) or normal placenta. This retrospective study reviewed 166 high-risk pregnant women who underwent MRI. Two radiologists independently assessed the presence of the seven established risk signs from the SAR-ESUR consensus and the novel IIMV sign. The IIMV sign was defined as continuous tubular or tortuous flow-void structures confined within the myometrium, associated with architectural distortion. Univariate logistic regression and interobserver agreement (kappa statistics) were used to select significant signs, which were then incorporated into a multivariate model to construct diagnostic combinations. AUCs were compared between combinations (with and without IIMV sign) in differentiating PI + PP group from PA + Normal groups. The novel IIMV sign demonstrated the highest specificity (0.91) and was independently associated with the differentiation between the PI + PP and Normal + PA groups. The optimal sign combination, which included IIMV, placental bulge, myometrial thinning, and bladder wall interruption, achieved an area under the curve (AUC) of 0.84. In contrast, the combination without IIMV showed a decreased AUC of 0.79 (*p* = 0.1203). The “increased intra-myometrial vascularity” sign is a highly specific marker for high-risk PAS. Its inclusion in an MRI-based diagnostic model improves the performance for identifying invasive placental disorders compared to combinations that do not utilize this novel sign.

**Supplementary Information:**

The online version contains supplementary material available at 10.1038/s41598-025-33179-0.

## Introduction

Placenta accreta spectrum (PAS) refers to the varying depths of abnormal attachment of chorionic villi to the decidua or myometrium and is classified into placenta accreta (PA), placenta increta (PI), and placenta percreta (PP)^1–4^. PAS is more likely to occur in cases involving placenta previa, previous cesarean delivery, prior uterine surgery (such as myomectomy, conization, or cervical cerclage), or in vitro fertilization^5^.

In patients with PA, the placenta can be detached by uterine contractions, manual or piecemeal removal of the placenta, massaging the uterus, etc.; whereas in patients with PI or PP, placental detachment requires special interventions or a multidisciplinary team approach to management for having risks of massive hemorrhage and myometrial damage of different area^6–9^.

Ultrasonography is still the first-line modality for antenatal diagnosis of PAS^10^. MRI is superior to characterize the placental topography with wider field of view and better soft tissue contrast^11,12^. A preoperative MRI could make appropriate recommendations such as prophylactic ureteral catheterization, the use of intraoperative blood salvage, and etc. to avoid or minimize complications^13^. Seven MRI signs, namely dark T2 bands, placental bulge, loss of low T2 retroplacental line, myometrial thinning, bladder wall interruption, focal exophytic mass, and abnormal vasculature of the placental bed are recommended for diagnosing PAS by the joint Society of Abdominal Radiology and European Society of Urogenital Radiology (SAR-ESUR) consensus^14^.

During routine MRI evaluations, we identified a novel imaging finding characterized by continuous tubular or tortuous flow-void structures within the myometrium, which we have designated as the “increased intra-myometrial vascularity (IIMV)” sign. In contrast to the physiological myometrial thinning observed in late-stage normal pregnancy, this vascular proliferation appears to be associated with architectural distortion in localized uterine regions, occasionally manifesting fusiform dilatation alterations, while demonstrating concomitant edematous hypertrophy of the corresponding myometrium. We hypothesized that it might be another MRI risk sign indicating the degree of abnormal circulation additional to abnormal vasculature of the placental bed^15,16^. To our knowledge, there has been no reports about the IIMV sign and its possible diagnostic value.

The purpose of this study was to investigate the value of IIMV and identify the performance of an appropriate MRI sign combination in differentiating PI or PP from PA or normal placenta.

## Materials and methods

### Patient selection

The institutional review board of Renji Hospital, Shanghai Jiao Tong University School of Medicine approved this retrospective study and waived the requirement for informed consent. All methods were performed in accordance with the relevant guidelines and regulations.

**Fig. 1:**
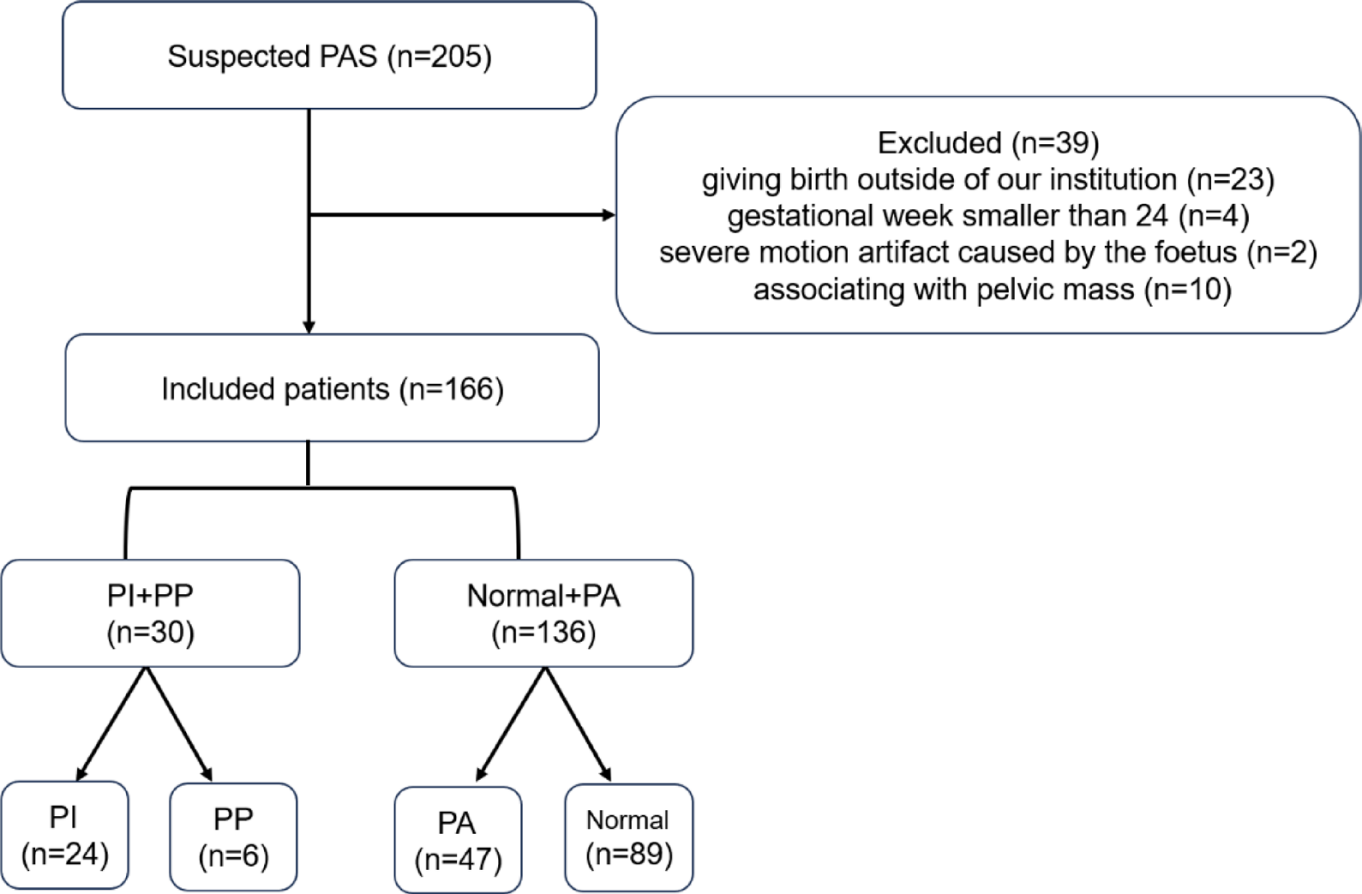
Flowchart of this study.

A total of 205 consecutive pregnant women at high-risk for PAS underwent MR imaging were retrospectively reviewed during July 2020 and July 2023. High-risk status was defined by the presence of one or more of the following criteria: placenta previa, prior cesarean delivery, history of other uterine surgery (e.g., myomectomy), or in vitro fertilization (IVF) pregnancy. Of them, 39 patients were excluded for the following reasons: (a) giving birth outside of our institution (resulting in lack of final diagnosis) (*n* = 23); (b) undergoing MRI examination before 24 gestational weeks because the recommended time for MRI assessment for PAS should be after 24 gestational weeks (*n* = 4)^17^; (c) having severe motion artifact caused by the foetus (*n* = 2); (d) associating with pelvic mass (*n* = 10: including uterine fibroids, *n* = 2; uterine fibroids accompanied by bilateral ovarian teratoma, *n* = 2; mass of Douglas pouch, *n* = 1; pelvic mass, *n* = 1; giant ovarian cyst (defined as a diameter ≥ 15 cm; *n* = 4)^18^. Finally, 166 patients were enrolled (Fig. 1).

Positive outcomes were defined as having PI or PP (*n* = 30, PI + PP group) as opposed to normal placenta or PA (*n* = 136, Normal + PA group).

Patient clinical characteristics, including maternal and gestational ages at MRI examination, number of caesarean deliveries, history of other direct surgery to the uterus, low-lying placenta or placenta previa, uterine artery embolization, and presence of major events were recorded. Major events included bleeding > 1000 ml, hysterectomy, bladder repairing, blood transfusion, the placement of an abdominal aortic balloon or uterine artery embolization, and an admission to the intensive care unit^1^.

### Standard of reference

In this study, the final diagnosis and classification of PAS were based on a combination of intraoperative findings and postoperative pathological results, in accordance with the FIGO guidelines.

### Diagnosis of PI or PP required meeting any one of the following criteria^5,19^

Intraoperative Criterion: After delivery of the fetus, the placenta failed to separate spontaneously, and manual removal was attempted but found to be exceptionally difficult or impossible due to dense adhesion between the placenta and the uterine wall, accompanied by uncontrollable excessive hemorrhage from the separation surface (e.g., blood loss > 1000 mL within a short period).

Pathological Criterion (for hysterectomy specimens): Histological examination confirmed that placental chorionic villi had invaded into the uterine myometrium (PI) or penetrated through the full thickness of the myometrium (PP), with an absence of the normal decidual layer at the site of attachment.

### Diagnosis of a normal placenta or placenta accreta (PA) required meeting any one of the following criteria^3,19^

Intraoperative Criterion: The placenta was delivered spontaneously or could be removed completely with gentle manual assistance, with controllable bleeding from the separation surface.

Pathological Criterion: Microscopic examination revealed either an intact decidual layer at the placental attachment site (Normal) or merely direct contact of chorionic villi with the myometrium without deep invasion (PA).

### MR image acquisition

MRI was performed using a 3-T MR unit (Ingenia, Philips Healthcare or MAGNETOM Prisma, Siemens Healthineers) with a 20-channel phased-array body coil. Patients were positioned supine with a partially full bladder. MRI protocols were generated according to SAR-ESUR guidelines’ recommendations^14^. T2-weighted imaging (T2WI) of the pelvis in the sagittal, coronal, and axial planes through the uterus is used to evaluate PAS disorders. Dixon sequence in the sagittal, coronal, and axial planes is used to detect any intra- or retroplacental hemorrhage/abruption. Details of MRI protocols were listed in Appendix 1. The entire MRI examination was completed within 30 min. No contrast media was used.

### Image interpretation

All 166 cases were separately reviewed by two abdominal radiologists (A, B with 4 and 14 years of experience, respectively) independently interpreted the T2WIs. They were aware about the study purpose and patients’ clinical information but were blinded to the final diagnosis, surgical records, and pathological findings. Furthermore, to further enhance the objectivity of our assessment, a third abdominal radiologist with 8 years of experience was invited for an independent review.

Prior to the formal review, both primary readers (A and B) participated in a calibration session using 10 more MRI cases of PAS (not included in the study cohort) to establish a consensus on the interpretation of all signs, particularly the novel ‘increased intra-myometrial vascularity’ sign. During the main study evaluation, cases with discrepant readings between Observers 1 and 2 were reviewed by the third, independent radiologist (Observer 3, with 8 years of experience), whose assessment served as the tie-breaker for the final analysis.

Each reader was asked to review the DICOM data in the axial, coronal, and sagittal planes of T2WIs for each case and record the presence or absence of a total of 8 risk signs based on the 7 signs recommended by SAR-ESUR consensus and the novel sign proposed in this study: (a) 7 signs recommended by SAR-ESUR consensus include bulge, loss of T2 hypointense interface, myometrial thinning, bladder wall interruption, intra-placental T2 dark bands, focal exophytic mass, abnormal vascularization of the placental bed; (b) the novel sign proposed in this study: increased intra-myometrial vascularity^14,20–22^.

**Fig. 2:**
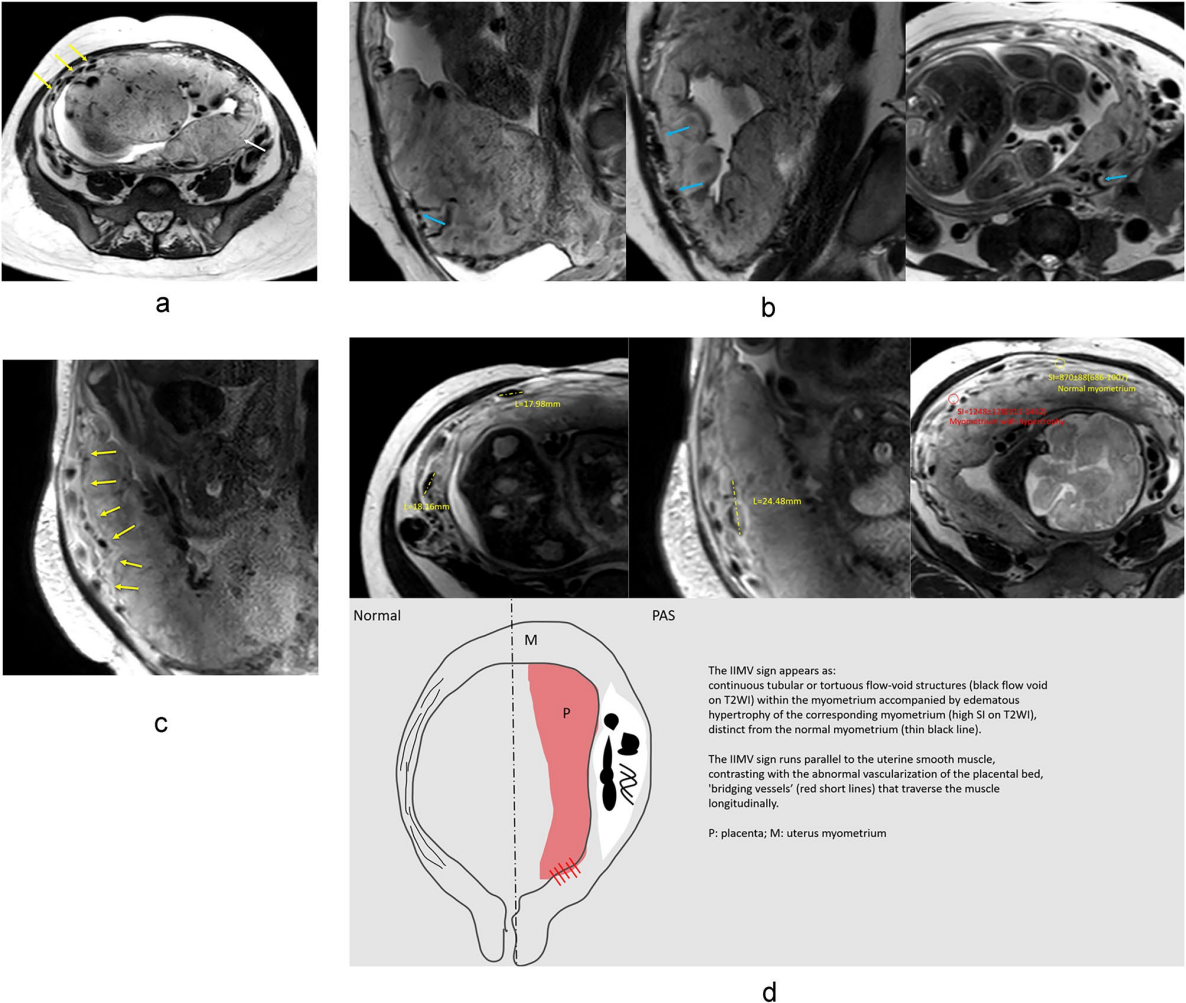
MRI appearance and Schematic diagram of the novel sign ‘increased intra-myometrial vascularity (IIMV)’. (**a**) The IIMV sign appears as continuous tubular or tortuous flow-void structures (yellow arrows) within the myometrium, distinct from the normal myometrium (white arrow). This phenomenon is associated with rich vascularization and fusiform dilatation in the lower uterine segments or the posterior wall area. This rich vascularization could cause localized myometrial elevation, sometimes accompanied by hypertrophy of the corresponding myometrium. (**b**) The IIMV sign runs parallel to the uterine smooth muscle, contrasting with the abnormal vascularization of the placental bed, ‘bridging vessels’ (blue arrows) that traverse the muscle longitudinally. (**c**) The IIMV sign (yellow arrows) appears to be associated with architectural distortion in localized uterine regions, manifesting fusiform dilatation alterations with concomitant hyperintense edematous hypertrophy of the corresponding myometrium on T2WI. (**d**) A schematic diagram summarizing the key features and distinctions of IIMV.

The novel sign, ‘increased intra-myometrial vascularity (IIMV),’ was defined as the presence of continuous tubular or tortuous flow-void structures *within the myometrium* on T2-weighted imaging, conceptually and morphologically distinct from the placental bed or subserosal vessels (Fig. 2a–c). To standardize assessment and enhance objectivity, a positive finding required meeting the following criteria (Fig. 2d): (1) the vascular structures must be clearly identifiable in at least two orthogonal planes (e.g., sagittal and axial); (2) on a single image slice, the continuous length of the vascular channel should exceed 15 mm; (3) the involved myometrium often demonstrated architectural distortion (e.g., fusiform bulging) and frequently exhibited increased T2 signal intensity relative to the adjacent normal myometrium, suggesting concomitant edema. A schematic diagram summarizing the key features and distinctions of this novel sign is provided in Fig. 2d.

IIMV presents as continuous tubular, tortuous, flow-void structures (small or large) confined within the myometrium in at least two planes, which remained relatively more prominent than those in neighbouring areas of the myometrium (Fig. 2a and d). Detailed definitions of 8 MRI risk signs are provided as Appendix 2. A positive finding was defined as the presence of the above signs in at least two planes. Normal placenta was defined as having homogeneous high signal intensity and smooth margins.

### Statistical analysis

The maternal and gestational ages at delivery, history of having caesarean section (number of times) were separately compared between PI + PP + Normal + PA groups using the Mann-Whitney test. The history of having other direct surgery to the uterus, placental site, lowing-lying placenta or placenta previa were compared between the two groups using the chi-square test.

The associations and discriminative ability of each MRI risk sign in differentiating PI + PP group from Normal + PA group were estimated using chi-square test and univariate logistic analysis. Receiver operating characteristic (ROC) analysis with area under the curve (AUC) by DeLong’s test was processed. Single odds ratio (OR) of 8 risk signs by univariate logistic regression analysis was recorded, respectively. Interobserver and intraobserver variability were assessed using the weighted *kappa* (k) value. A k value of < 0.20 was interpreted as slight agreement, 0.21–0.40 fair agreement, 0.41–0.60 as moderate agreement, 0.61–0.80 as substantial agreement, and > 0.81 as almost perfect agreement^5,15,23^.

In case of discordance, a lower AUC was selected. A k value > 0.20 and a selected AUC > 0.60 were considered statistically significant MRI signs. These signs were further selected for multivariate logistic regression analysis to construct a sign combination. The sensitivity, specificity, positive predictive value (PPV), negative predictive value (NPV) of sign combinations, and adjusted ORs were computed from multivariate logistic regression analysis.

A pairwise comparison of the AUC was conducted to assess the performance of our sign combination with the novel sign IIMV versus the combination excluding it in differentiating the PI + PP group from the Normal + PA group. The Akaike information criterion (AIC) was used for model comparison, with lower AIC values indicating a superior and more stable model.

Statistical analyses were performed using JMP Pro 15 (SAS Institute, Cary, NC). A p value < 0.05 was considered statistically significant.

## Results

Overall, 166 patients enrolled in the present study. For the 30 patients in PI + PP group, 24 were diagnosed with PI and 6 with PP; while for the 136 people in Normal + PA group, 47 were diagnosed with PA and the remaining 89 were normal.

Significant differences were found in history of caesarean section, low-lying placenta or placenta previa, receiving uterine artery embolization, the presence of major events, and bleeding > 1000 ml between the two groups (*p* = 0.0003, 0.0091, < 0.0001, <0.0001, and < 0.0001, respectively). No other significant difference was observed in other patients’ demographics (Table 1).

**Table 1:** Patients’ demographics between Normal + PA and PI + PP groups.

Variables		Normal + PA (*n* = 136)	PI + PP (*n* = 30)	*p* value
Maternal age at delivery (years) (Mean ± SD)		34 ± 3.95	36 ± 4.95	0.0511
Gestational age at MRI (weeks) (Mean ± SD)		34 ± 3.00	32 ± 3.93	0.0519
History of cesarean section (number of times (%))	0	82 (60%)	8 (27%)	0.0003*
	1	49 (36%)	16 (53%)	
	2	4 (3%)	6 (20%)	
	3	1 (100%)	0 (0%)	
History of other direct surgery to the uterus (N (%))		18 (82%)	4 (18%)	0.9886
IVF (N (%))		18 (82%)	18 (82%)	0.1470
GDM (N (%))		29 (21%)	9 (30%)	0.3059
Hypertension (N (%))		7 (5%)	2 (7%)	0.7394
Low-lying placenta or placenta previa (N (%))		110 (82%)	30 (100%)	0.0091*
Postpartum Interventions (N (%))		31 (23%)	18 (60%)	< 0.0001*
Major events (N (%))		20 (15%)	27 (90%)	< 0.0001*

p values with * are statistically significant.

PA: placenta accrea, PI: placenta increta, PP: placenta percreta.

The direct surgery included myomectomy, conization, dilation and curettage, cervical cerclage.

IVF: in vitro fertilization.

GDM: gestational diabetes mellitus.

Postpartum Interventions: refers to uterine artery embolization.

Major events included bleeding > 1 L, hysterectomy, bladder repairing, blood transfusion, the placement of an abdominal aortic balloon or uterine artery embolization, and an admission to the intensive care unit.

The IIMV sign showed a sensitivity of 0.67, a specificity of 0.91, a positive predictive value (PPV) of 0.67, and a negative predictive value (NPV) of 0.78, as detailed in Table 2 (data derived from Observer 1). There were 10 false-negative cases of IIMV (placenta increta, *n* = 10) and 30 false-positive cases (placenta accreta, *n* = 13, normal, *n* = 17).

**Table 2:** Discriminative ability of each sign in differentiating PI + PP from Normal + PA groups.

MRI risk signs (*N* (%))	Observer 1				Observer 2				k value
	Normal + PA (*n* = 136)	PI + PP (*n* = 30)	p1	AUC1	Normal + PA (*n* = 136)	PI + PP (*n* = 30)	p2	AUC2	
Increased intra-myometrial vascularity	30 (22%)	20 (67%)	< 0.0001*	0.72	53 (38%)	18 (60%)	0.0289*	0.61	0.28
Bulge†	92 (68%)	30 (100%)	< 0.0001*	0.67	72 (53%)	25 (83%)	0.0022*	0.65	0.23
Loss of T2 hypointense interface†	102 (75%)	29 (97%)	0.0023*	0.61	69 (51%)	21 (70%)	0.0552	0.59	0.20
Myometrial thinning†	78 (57%)	25 (83%)	0.0053*	0.63	27 (20%)	16 (53%)	0.0002*	0.67	0.22
Bladder wall interruption†	11 (8%)	13 (43%)	< 0.0001*	0.68	4 (3%)	10 (33%)	< 0.0001*	0.65	0.41
Abnormal vascularization of the placental bed†	56 (41%)	19 (63%)	0.0273*	0.61	29 (21%)	12 (40%)	0.0337*	0.59	0.21
T2 dark band†	130 (96%)	29 (97%)	0.7909	0.51	68 (50%)	17 (57%)	0.5085	0.53	0.15
Focal exophytic mass†	6 (4%)	6 (20%)	0.0084*	0.58	6 (4%)	6 (20%)	0.0028*	0.58	0.36

†: signs recommended by SAR-ESUR guidelines.

AUC: area under the curve.

The *k* value ranged from 0.15 to 0.41 among 8 MRI risk signs, which showed a fair to moderate agreement between Observer 1 and Observer 2 (Table 2). The results of intraobserver and interobserver agreement statistics (data derived from Observer 2 and Observer 3) are provided in Appendix 4 and Appendix 5 and corroborate the initial findings. A higher level of agreement was observed between the two senior radiologists than between the senior and junior radiologists.

Bulge, myometrial thinning, IIMV, and bladder wall interruption showed significant correlation (with kappa values > 0.20 and AUCs > 0.60) in distinguishing between the PI + PP and Normal + PA groups (Table 2). Single odds ratio of 8 risk signs in differentiating PI + PP from Normal + PA groups was presented in Appendix 3.

Multiple logistic regression analysis revealed that bulge (*p* = 0.0011), IIMV (*p* = 0.0026), and bladder wall interruption (*p* = 0.0081) were independently associated with an increased likelihood of PI or PP (Table 3).

**Table 3:** Diagnostic performance of sign combinations in differentiating PI + PP from Normal + PA groups.

Sign combination	Selected risk signs	*p* value	Odds ratio	AUC	AIC	SE	SP	PPV	NPV	Ac
Our combination	Bulge	0.0011	10.70	0.84	122.02	0.70	0.76	0.49	0.92	0.75
	Myometrial thinning	0.2061	1.60							
	Increased intra-myometrial vascularity	0.0026	9.10							
	Bladder wall interruption	0.0081	7.02							
Combination without the novel sign	Bulge	0.0003	12.86	0.79	128.44	0.87	0.54	0.29	0.94	0.59
	Myometrial thinning	0.2131	1.55							
	Bladder wall interruption	0.0005	11.94							

SE: sensitivity; SP: specificity; PPV: positive predictive value; NPV: negative predictive value; Ac: accuracy; AUC: area under the curve; AIC: Akaike information criterion; p values < 0.05 are statistically significant.

Our sign combination, which included the novel IIMV sign along with bulge, myometrial thinning, and bladder wall interruption, achieved a diagnostic performance of 0.84 (Table 3). A pairwise comparison of AUCs revealed a decreased performance when the IIMV sign was excluded, with AUCs of 0.84 and 0.79 respectively (*p* = 0.1203). The AIC was lower with our sign combination, indicating its superior model fit when compared to the combination excluding the IIMV sign.

## Discussion

Placenta accreta spectrum (PAS) is a condition where placental tissue abnormally adheres to or penetrates the uterine wall, often at a scar site. It is categorized into placenta accreta (PA), placenta increta (PI), and placenta percreta (PP), with PI and PP posing higher risks of hemorrhage and myometrial damage, necessitating specialized management^24^. Accurate prenatal diagnosis of high-risk PAS is vital for maternal safety and can mitigate severe complications. Our study evaluated the diagnostic efficacy of 8 MRI risk signs, including the novel ‘increased intra-myometrial vascularity (IIMV)’ sign identified in this research, to differentiate high-risk PAS (PI + PP) from low-risk PAS (Normal + PA) groups, as different treatments may be necessary.To the best of our knowledge, this is the first study to report increased intra-myometrial vascularity within the uterine myometrium observed on MRI, in association with high-risk PAS and major bleeding events.

**Fig. 3:**
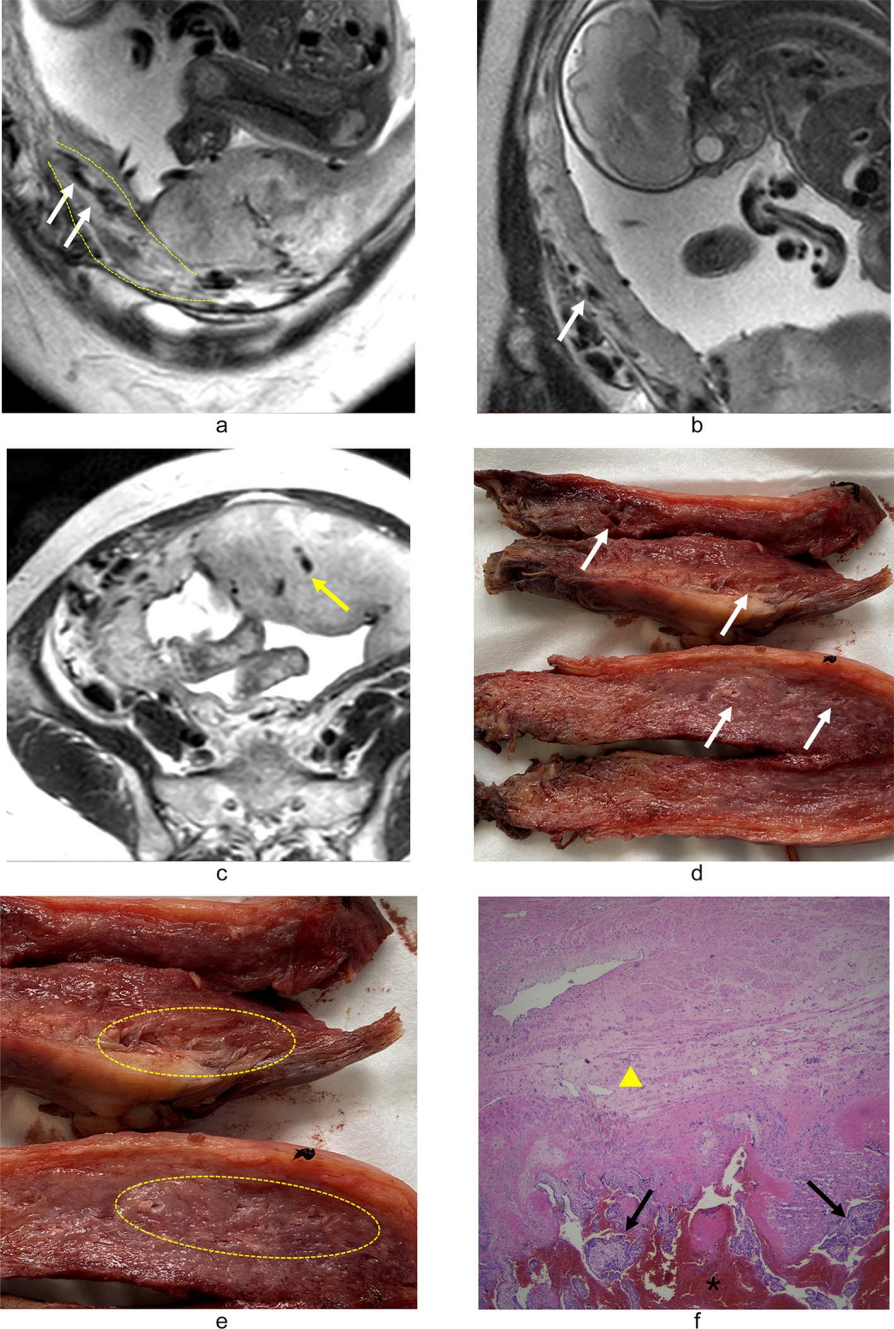
A 37-year-old woman with placenta previa. Obstetric ultrasound suggested a high risk for PAS. Coronal (**a**, **b**) and axial (**c**) planes showed multiple enlarged flow-void signal within the myometrium (white arrows), and the localized myometrium with hypertrophy showed a fusiform-shaped morphology (yellow dashed lines). Abnormal vascularization in the deep parenchyma was found (yellow arrow) (**c**). Bilateral internal iliac artery balloon was preoperatively performed. Hysterectomy specimen revealed that multiple increased vascularity within the myometrium (white arrows) (**d**, **e**). Rich vascularization can also be found under the serosa of the uterus (yellow dashed area) (**e**). Surgical findings revealed that the lower segment of the uterus was thin and replaced by rich vascularity, and some of the placenta tightly invaded the lower segment and the bladder, and the lower uterine segment was not contracting well. The total intraoperative bleeding was 3600 ml, and she was diagnosed as PP by the histopathology (**f**).

**Fig. 4:**
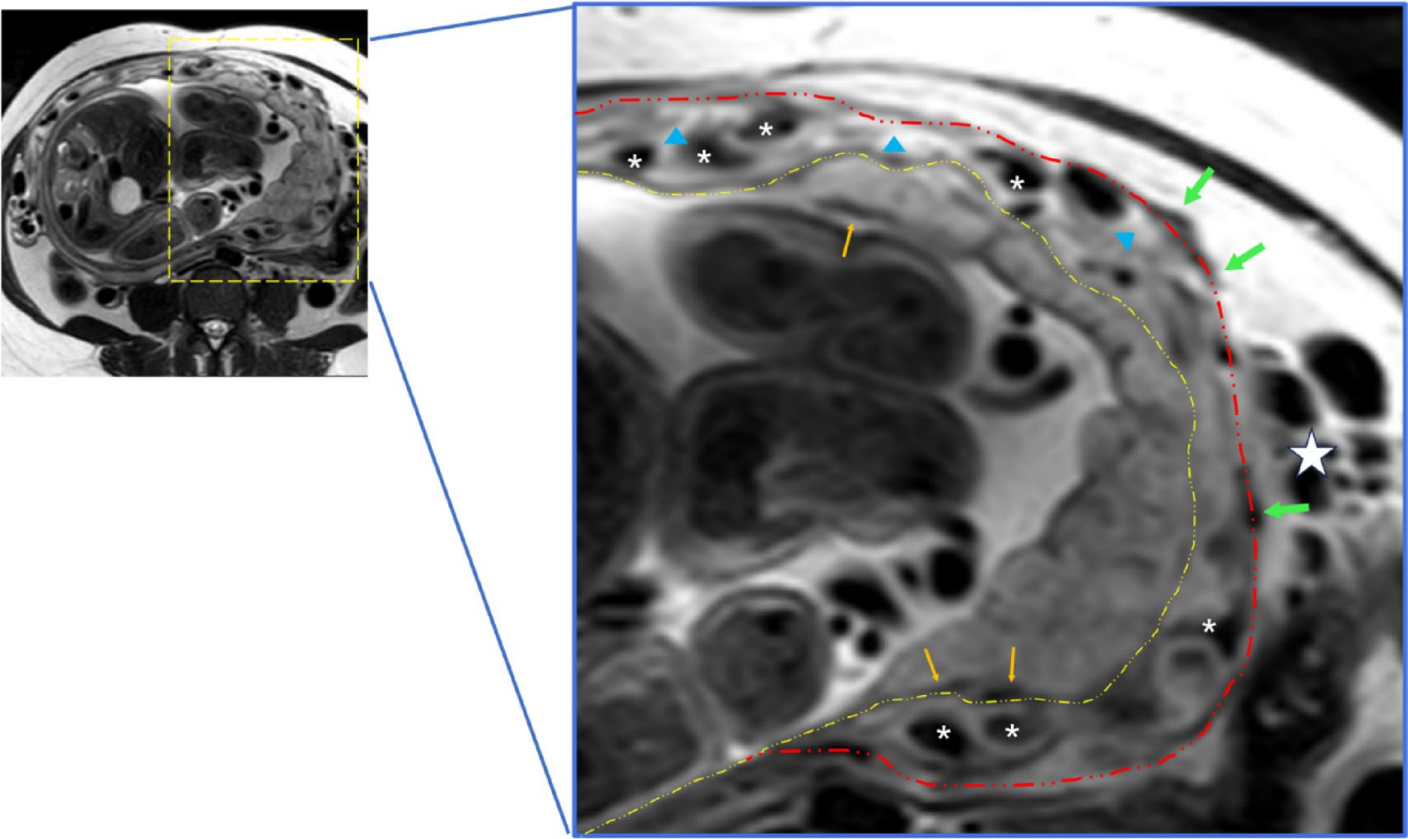
The key differences between the Novel Sign and abnormal vascularization of the placental bed. The enhanced vascularization confined within the myometrium can be observed, which are distinct from the placental bed and parametrial vessels. White asterisks: the novel sign ‘increased intra-myometrial vascularity’ appears as continuous tubular or tortuous flow-void structures within the myometrium. Myometrium with hypertrophy, indicated by blue triangles, exhibits higher T2 signal intensity compared to myometrium without hypertrophy. Orange arrows: bed vascularization. Green arrows: serosa vascularization. Yellow dashed line: placenta-utero interface. Red dashed line: serosa side.

In our clinical MRI practice, we identified a distinct vascular pattern characterized by continuous tubular or tortuous flow-void structures within the myometrium of placenta increta/percreta (PI/PP) cases (Figs. 3a–e and 4). These vascular channels demonstrate three critical morphological features: (1) dense vascular networks with fusiform luminal dilatation predominantly in the lower uterine segment/posterior wall regions; (2) parallel orientation to uterine smooth muscle fibers, distinct from the longitudinal bridging vessels described in prior studies (Figs. 2b and d and 4)^25^; (3) spatial correlation with myometrial architectural disarray. This angioarchitectural rearrangement may contribute to impaired placental detachment through two potential mechanisms: (a) mechanical interference from hypertrophied vascular complexes, and (b) compromised decidual-myometrial interface integrity secondary to stromal edema.

Similar to the placental T2 dark band, infarction, and recess, each of these parenchymal signs reflects the pathophysiological processes associated with PAS^15^. As the placentation becomes more aggressive, the uteroplacental vasculature changes become more pronounced; deeper invasion of the trophoblast into the myometrium and infiltration of chorionic villi into myometrial vascular spaces have recently been reported in PI and PP^19,20,26,27^. The IIMV sign might be another risk sign indicating the underlying progression of abnormal circulation, in addition to intra-placental vascularity and vascularization of the placental bed. The evaluation of placental vasculature may need more detailed information to accurately reflect the overall vascular dynamics^28,27^.

We also noticed that IIMV, often accompanied by fusiform dilatation, may be correlated to hypoxia and neoangiogenesis in PAS-affected pregnancies, particularly in the second and third trimesters^4,27,29^. This hypoxic state potentially accelerates smooth muscle cell edema (Fig. 2d: the signal intensity of the normal myometrium area on T2WI was lower than that of myometrium with edema), leading to the replacement of contractile myometrial structures with non-contractile, dilated vessels lacking tunica media, which results in weak contractions and placental retention^26,27,29^. Radiologists need to evaluate intra-myometrial vascularity by considering both flow-void signal intensity and myometrial shape distortion, rather than relying solely on flow-void signal intensity as with intra-placental vascularity and placental bed vascularization.

Interestingly, in the current study, IIMV was the most effective risk sign of vascularization for identifying invasive placenta, with a single AUC ranging from 0.61 to 0.72, the specificity and NPV was 0.91and 0.78, respectively. The frequency and single ORs of IIMV (67%; 4.72 ~ 7.07) were higher than that of “abnormal vascularization of the placental bed” (63%; 2.44 ~ 2.47) in patients with high-risk PAS (PI + PP group) (Table 2, Appendix 3).

**Fig. 5:**
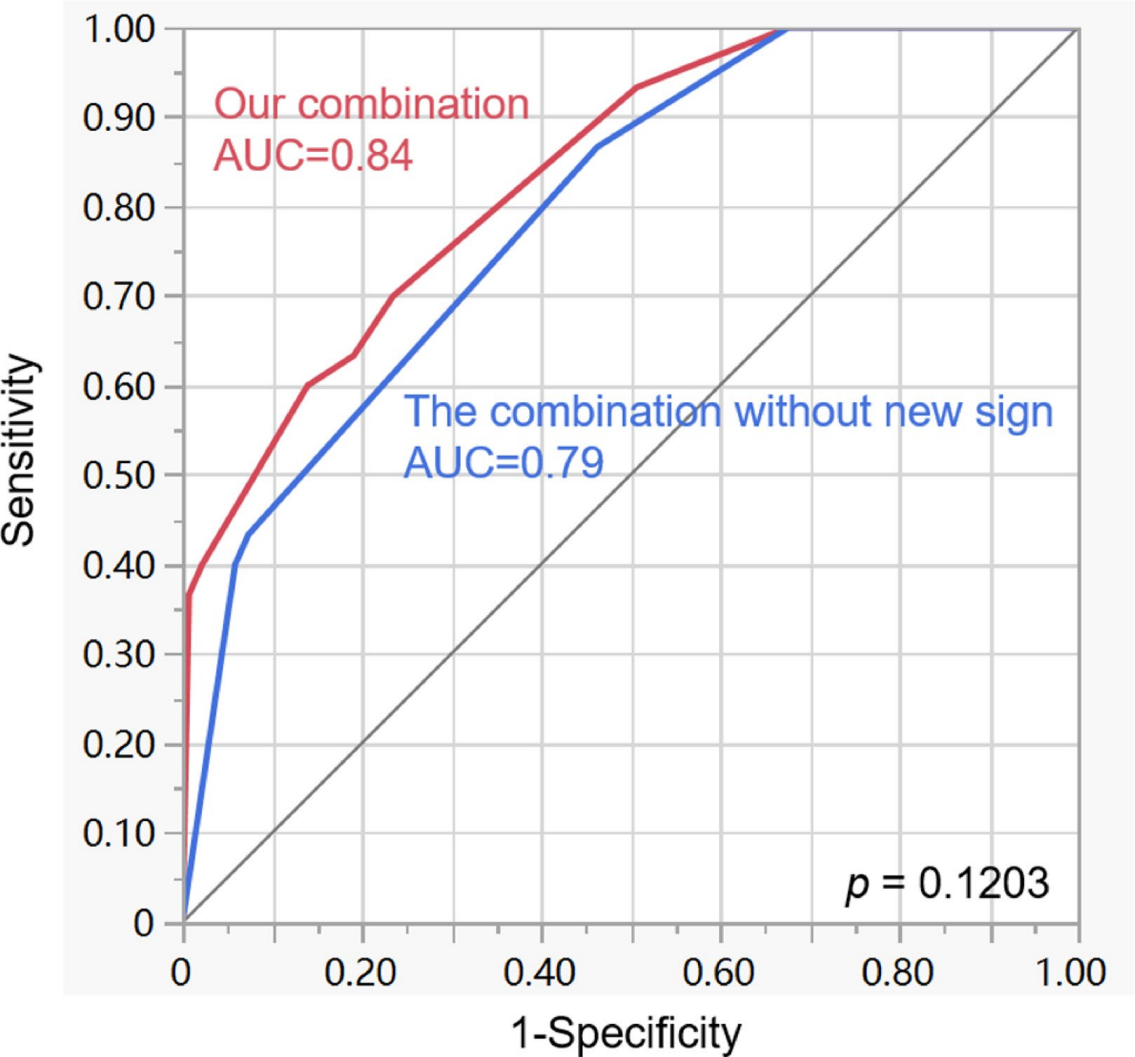
ROC curves comparing diagnostic combinations with and without the novel IIMV sign. Graph depicting the receiver operating characteristic (ROC) curve analysis using areas under the curve (AUCs) for our combination and the combination excluding the novel sign ‘increased intra-myometrial vascularity’ for differentiating between PI + PP group and the Normal + PA group. A comparison of AUCs shows that the diagnostic performance of our combination is better than that of the combination excluding the novel sign (0.84 vs. 0.79, *p* = 0.1203).

Our sign combination, which included the novel sign ‘IIMV’ along with bulge, myometrial thinning, and bladder wall interruption, achieved a diagnostic performance of 0.84 and a specificity of 0.79 (Table 3). A pairwise comparison of AUCs revealed a decreased performance when the ‘increased intra-myometrial vascularity’ sign was excluded, with AUCs of 0.84 and 0.79 respectively (*p* = 0.1203) (Fig. 5). Although no significant difference was found between the two groups, this result suggested the potential added value of IIMV in differentiating the PI + PP group from the Normal + PA group^30^. By integrating this novel sign into preoperative assessments, radiologists can more accurately determine the degree of abnormal vascularization, providing an estimate of PAS severity and an indication of the potential scale of clnical bleeding.

The high specificity of IIMV is clinically relevant. Its identification on preoperative MRI could signal the need for heightened preparedness, such as assembling a multidisciplinary team, ensuring blood product availability, or considering prophylactic vascular interventions. This sign may help stratify patients within the PAS spectrum, potentially guiding the extent of surgical intervention.

The similar frequency of IIMV and “abnormal vascularization of the placental bed” may be explained by fact that the limited anatomical space within the myometrium and patients with PI or PP in our study who showed increased intra-myometrial vascularity to some degree had concomitant abnormal vascularization of the placental bed^31,32^. Additionally, we need to acknowledge that this association could have potentially influenced the p value in pairwise comparison of AUCs to some degree.

This study has several limitations. First, the sample size of our high-risk PI + PP group (n = 30), and particularly the PP subgroup (n = 6), remains limited, a common challenge in studying this severe but relatively rare condition. This may have impacted the statistical power of our subgroup analyses and the AUC comparison, potentially contributing to the non-significant p-value (p = 0.1203) observed between the model with and without IIMV, despite a clinically relevant improvement in AUC (0.84 vs. 0.79). We acknowledge that this limits the immediate generalizability of our findings. Future multi-center prospective studies with larger cohorts are strongly warranted to validate the diagnostic value of IIMV and our proposed sign combination. Second, the evaluation of MRI signs, including our novel ‘IIMV’ inherently involves a degree of subjective visual interpretation. Although we integrated assessment of the corresponding myometrium—evaluating its shape and signal intensity—to enhance standardization and demonstrated fair to moderate interobserver agreement, the potential for variability remains. This variability is further contextualized by the differing experience levels of our two primary readers with 4 and 14 years, a factor that reflects the real-world clinical environment where radiologists with varied expertise collaborate. It is noteworthy, however, that the significant interobserver agreement achieved across this experience gap underscores the sign’s recognizability. Furthermore, the involvement of a third, independent radiologist and the analysis of intraobserver variability (provided in the Appendix 4) strengthen the reliability of our findings. Future studies would greatly benefit from the development and application of quantitative, computer-aided diagnostic such as vessel segmentation and flow quantification algorithms—to objectively measure vascular patterns and minimize observer-dependent bias. Third, an inherent limitation that needs to be determined is that discrepancies exist between surgical findings and histopathologic findings, which may considerably underestimate the degree of PAS^33,34^.

## Conclusion

The “increased intra-myometrial vascularity (IIMV)” sign is a highly specific marker for high-risk PAS. Its inclusion in an MRI-based diagnostic model improves the performance for identifying invasive placental disorders compared to combinations that do not utilize this novel sign.

## Supplementary Information

Below is the link to the electronic supplementary material.

Supplementary Material 1

## Data Availability

Data available on request from the authors, please contact Hainan Ren, the first author, the email is [merylren1994@163.com](mailto: merylren1994@163.com) .

## Abbreviations

PAS: Placenta accreta spectrum
PA: Placenta accreta
PI: Placenta increta
PP: Placenta percreta
MRI: Magnetic resonance imaging
ROC: Receiver operating characteristic curve analysis
AUC: Area under the curve
