# Supplementary material for: Increased intra-myometrial vascularity adds diagnostic value to MRI for high-risk placenta accreta spectrum
Source: Sci Rep. 2025 Dec 22;15:45134. doi: 10.1038/s41598-025-33179-0 (PMC12749984; doi:10.1038/s41598-025-33179-0)
Supplement: Supplementary file 1 — Supplementary Material 1 [file 41598_2025_33179_MOESM1_ESM.docx]

Appendix 1

Sequence index of Ingenia, Philips Healthcare

| Sequence | Orientation | TR/TE (ms) | Thickness(mm) | FOV (mm) | Matrix |
| --- | --- | --- | --- | --- | --- |
| SSh-FSE | coronal | 2000/100 | 4 | 380 | 316*251 |
| SSh-FSE | sagittal | 2000/100 | 4 | 380 | 316*251 |
| SSh-FSE | axial | 2000/100 | 4 | 350 | 252*221 |
| bFFE | coronal | 3.1/1.55 | 4 | 400 | 268*204 |
| bFFE | sagittal | 2.7/1.36 | 4 | 400 | 268*205 |
| mDixon | axial | 3.6/1.31 | 4 | 400 | 252*196 |

T2w SSh-FSE: T2-weighted single shot Fast Spin Echo

bFFF: balanced fast field echo

TR/TE: repetition time/echo time

FOV: field of view

mDixon: modified Dixon sequence

Sequence index of MAGNETOM Prisma, Siemens Healthineers

| Sequence | Orientation | TR/TE (ms) | Thickness(mm) | FOV (mm) | Matrix |
| --- | --- | --- | --- | --- | --- |
| HASTE | sagittal | 868/150 | 4 | 400 | 256*320 |
| HASTE | coronal | 868/150 | 4 | 400 | 256*384 |
| HASTE | axial | 776/150 | 4 | 400 | 256*320 |
| T1 VIBE Dixon | axial | 3.97/1.23/2.46 | 3 | 400 | 189*320 |

HASTE: Half-Fourier Acquisition Single-shot Turbo spin Echo imaging

TR/TE: repetition time/echo time

FOV: field of view

T1 VIBE Dixon : T1 Volumetric Interpolated Breath-hold Examination Dixon

Appendix 2

Detailed definitions of 8 MRI signs

| Signs | Definition |
| --- | --- |
| Bulge† | Deviation of the uterine serosa from the expected plane caused by abnormal bulge of placental tissue toward adjacent organs, typically toward the bladder and parametrium |
| Loss of T2 hypointense interface† | The loss of a thin dark line behind the placental bed, as seen on T2-weighted images |
| Myometrial thinning† | Thinning of the myometrium over the placenta to less than 1 mm or even invisible |
| Bladder wall interruption† | Irregularity or disruption of the normal hypointense bladder wall, which can be accompanied by blood products in the bladder lumen |
| T2 dark band† | One or more areas of hypointensity on T2W images manifesting like linear in configuration and in contact with the maternal surface of the placenta |
| Focal exophytic mass† | Defined as placental tissue seen protruding through the uterine wall and extending beyond it |
| Abnormal vascularization of the placental bed† | Prominent vessels in the placental bed with disruption of the uteroplacental interface |
| Increased intra-myometrial vascularity* | Continuous tubular, tortuous, flow-void (small or large) structures within the myometrium accompanying with spindle-shaped dilatation of the corresponding lower uterine segments or the posterior wall area |

†: signs recommended by SAR-ESUR guidelines

*: the new sign proposed in this study

Appendix 3

Single odds ratio of 8 risk signs in differentiating PI+PP from Normal+PA groups by chi-square test

| MRI risk signs | ORs of observer 1 | ORs of observer 2 |
| --- | --- | --- |
| Bulge† | 3.81 | 4.44 |
| Loss of T2 hypointense interface† | 9.60 | 2.01 |
| Myometrial thinning† | 3.72 | 4.61 |
| Bladder wall interruption† | 8.69 | 16.5 |
| T2 dark band† | 1.34 | 1.31 |
| Focal exophytic mass† | 5.41 | 5.42 |
| Abnormal vascularization of the placental bed† | 2.47 | 2.44 |
| Increased intra-myometrial vascularity* | 7.07 | 4.72 |

†: signs recommended by SAR-ESUR guidelines

*: the new sign proposed in this study

Appendix 4

Intraobserver agreement for MRI signs in differentiating high-risk (PI/PP) from low-risk (Normal/PA) placenta accreta spectrum.

| MRI risk signs  (N (%)) | Weighted κ (95% CI) | Asymptotic Standard Error | z-statistic | p value |
| --- | --- | --- | --- | --- |
| **Increased intra-myometrial vascularity*** | 0.583 (0.339-0.826) | 0.124 | 4.381 | <0.001 |
| Bulge† | 0.373 (0.072-0.675) | 0.153 | 3.540 | <0.001 |
| Loss of T2 hypointense interface† | 0.253 (0.004-0.509) | 0.131 | 2.253 | 0.024 |
| Myometrial thinning† | 0.316 (0.060-0.573) | 0.131 | 2.584 | 0.010 |
| Bladder wall interruption† | 0.489 (0.155-0.823) | 0.170 | 4.183 | <0.001 |
| Abnormal vascularization of the placental bed† | 0.656 (0.447-0.864) | 0.106 | 4.991 | <0.001 |
| T2 dark band† | 0.292 (0.066-0.649) | 0.182 | 3.075 | 0.002 |
| Focal exophytic mass† | 0.706 (0.363-1.049) | 0.175 | 5.992 | <0.001 |

PI, placenta increta; PP, placenta percreta; PA, placenta accreta; κ, kappa coefficient.
*Novel sign proposed in this study.
†Sign recommended by the SAR-ESUR (Society of Abdominal Radiology and European Society of Urogenital Radiology) consensus guidelines.

Appendix 5

Interbserver agreement for MRI signs in differentiating high-risk (PI/PP) from low-risk (Normal/PA) placenta accreta spectrum between observer 2 and 3

| MRI risk signs  (N (%)) | Weighted κ (95% CI) | Asymptotic Standard Error | z-statistic | p value |
| --- | --- | --- | --- | --- |
| **Increased intra-myometrial vascularity*** | 0.762 (0.572-0.867) | 0.115 | 4.216 | <0.001 |
| Bulge† | 0.214 (0.146-0.353) | 0.127 | 0.924 | 0.209 |
| Loss of T2 hypointense interface† | 0.340 (0.184-0.463) | 0.139 | 1.406 | 0.160 |
| Myometrial thinning† | 0.309 (0.072-0.547) | 0.121 | 2.371 | 0.018 |
| Bladder wall interruption† | 0.842 (0.716-0.912) | 0.184 | 4.982 | <0.001 |
| Abnormal vascularization of the placental bed† | 0.474 (0.056-0.707) | 0.137 | 2.146 | 0.016 |
| T2 dark band† | 0.570 (0.431-0.798) | 0.116 | 4.327 | <0.001 |
| Focal exophytic mass† | 0.539 (0.173-0.743) | 0.285 | 2.579 | 0.010 |

PI, placenta increta; PP, placenta percreta; PA, placenta accreta; κ, kappa coefficient.
*Novel sign proposed in this study.
†Sign recommended by the SAR-ESUR (Society of Abdominal Radiology and European Society of Urogenital Radiology) consensus guidelines.
